# Evaluation of a birth preparation program on lumbopelvic pain, urinary incontinence, anxiety and exercise: a randomized controlled trial

**DOI:** 10.1186/1471-2393-13-154

**Published:** 2013-07-29

**Authors:** Maria Amélia Miquelutti, José Guilherme Cecatti, Maria Yolanda Makuch

**Affiliations:** 1 Department of Obstetrics and Gynecology, School of Medical Sciences, University of Campinas (UNICAMP), Caixa Postal 6181 13083-970, Campinas, São Paulo, Brazil; 2 Center for Research on Reproductive Health of Campinas (Cemicamp), Campinas, SP, Brazil

**Keywords:** Antenatal exercises, Birth preparation program, Urinary incontinence, Low back pain, Pelvic pain, Anxiety, Pregnancy

## Abstract

**Background:**

Antenatal preparation programmes are recommended worldwide to promote a healthy pregnancy and greater autonomy during labor and delivery, prevent physical discomfort and high levels of anxiety. The objective of this study was to evaluate effectiveness and safety of a birth preparation programme to minimize lumbopelvic pain, urinary incontinence, anxiety, and increase physical activity during pregnancy as well as to compare its effects on perinatal outcomes comparing two groups of nulliparous women.

**Methods:**

A randomized controlled trial was conducted with 197 low risk nulliparous women aged 16 to 40 years, with gestational age ≥ 18 weeks. Participants were randomly allocated to participate in a birth preparation programme (BPP; n=97) or a control group (CG; n=100). The intervention was performed on the days of prenatal visits, and consisted of physical exercises, educational activities and instructions on exercises to be performed at home. The control group followed a routine of prenatal care. Primary outcomes were urinary incontinence, lumbopelvic pain, physical activity, and anxiety. Secondary outcomes were perinatal variables.

**Results:**

The risk of urinary incontinence in BPP participants was significantly lower at 30 weeks of pregnancy (BPP 42.7%, CG 62.2%; relative risk [RR] 0.69; 95% confidence interval [CI] 0.51-0.93) and at 36 weeks of pregnancy (BPP 41.2%, CG 68.4%; RR 0.60; 95%CI 0.45-0.81). Participation in the BPP encouraged women to exercise during pregnancy (p=0.009). No difference was found between the groups regarding to anxiety level, lumbopelvic pain, type or duration of delivery and weight or vitality of the newborn infant.

**Conclusions:**

The BPP was effective in controlling urinary incontinence and to encourage the women to exercise during pregnancy with no adverse effects to pregnant women or the fetuses.

**Trial registration:**

Clinicaltrials.gov, (NCT01155804)

## Background

Antenatal preparation programmes involve several techniques and activities performed by different healthcare professionals. The main aims of antenatal preparation programmes are to promote healthy practices, minimize excessive anxiety and prevent or minimize the discomforts of pregnancy and labor. Such programmes may include educational activities, physical exercise and psychoprophylactic techniques, among others. Despite the fact that such programmes are recommended in different parts of the world, Gagnon and Sandall in a systematic review found no evidence regarding the benefits of general antenatal education for childbirth [1].

The practice of regular physical exercise during pregnancy with the objective of keeping women healthy during pregnancy is recommended by the American College of Obstetricians and Gynecologists (ACOG) [2], and can be included in antenatal programs. Moreover, daily exercises can prevent gestational diabetes and excessive gestational weight gain [2-4]. There is evidence that physical exercise during pregnancy does not increase the risk of muscle injuries or changes in arterial blood pressure [5], and does not increase the risk of preterm labor or low fetal weight [3,6-8].

Investigations have been conducted to evaluate whether exercise during pregnancy is able to reduce discomfort resulting from lumbopelvic pain and decrease the occurrence of urinary incontinence. Pennick and Young [9] in a systematic review showed evidence that the practice of specific exercises was able to alleviate lumbopelvic pain during pregnancy. However, it is still under debate whether pelvic floor muscle exercises during pregnancy could protect against urinary incontinence both during pregnancy and in the postpartum period [10-12].

Anxiety during pregnancy has been related to adverse outcomes, such as fetal distress, premature labor, low birth weight, and problems in child development [13-16]. Khianman et al. [16], in a systematic review discussed how the use of relaxation techniques (breathing exercises, massage, yoga, reflexology, visualization) during pregnancy may reduce stress and anxiety.

Information related to the effect of antenatal preparation programmes on perinatal outcomes is scarce. Some studies have suggested that a shorter duration of labor and a reduction in Cesarean section (C-section) rates is important to improve perinatal outcomes [17,18]. The existing evidence is mainly based on small studies that analysed outcomes in women from middle or low income countries with high C-section rates.

The objective of this study was to evaluate the effectiveness and safety of a birth preparation programme (BPP) to minimize lumbopelvic pain, urinary incontinence, anxiety, and increase physical activity during pregnancy, as well as, to compare its effects on perinatal outcomes comparing two groups of nulliparous women.

## Methods

A prospective, randomised, controlled clinical trial was conducted at the Women’s Integral Health Care Hospital (CAISM), University of Campinas (UNICAMP) and four municipal primary healthcare centers in Campinas, São Paulo between June 2009 and September 2011. The study was approved by the Institutional Review Board. The eligible women were recruited at the same time as the first data collection was performed and after the explanation about the study they had a time to consider participation or not. Those who agreed to participate gave their written consent prior to being included in the study.

Primary outcomes were: lumbopelvic pain, urinary incontinence (UI), anxiety, and physical activity performed at home.

### Sample size

Sample size was calculated based on the occurrence of lumbar pain in a previous study (66% in the control group and 46% in the intervention group; n=192) [18]. The primary outcomes considered for sample size estimation were urinary incontinence, lumbopelvic pain, and anxiety; however lumbar pain presented the lowest variation and therefore the highest sample to be studied. A proportion of one intervention for each control was determined [1:1 ratio], with a significance level of 5% and an 80% power of the test, resulting in 192 subjects to be randomized into two groups. Considering the possibility of post-randomization exclusions, 205 women were randomized.

### Patients and eligibility

The inclusion criteria were: pregnant women with a single fetus, age 16 to 40 years and gestational age 18 to 24 weeks. The exclusion criteria were: pathological conditions prior to pregnancy (heart conditions, diabetes, hypertension, bronchitis, asthma, HIV positive); pathological conditions of the pregnancy (gestational hypertension, gestational diabetes, and preeclampsia), contraindications to the practice of physical activity (persistent bleeding, preterm labor, incompetent cervix, acute febrile infection, and fetal growth restriction) or indication for elective C-section (placenta previa, cephalopelvic disproportion).

### Patient evaluation

Data collection during pregnancy was performed on the days of medical visits at: 18–24 weeks of pregnancy at admission to the study; 28–30 weeks of pregnancy (intermediate evaluation) and at 36–38 weeks of pregnancy (final evaluation). Anxiety was evaluated through the state-trait anxiety inventory (STAI), validated for use in Brazil [19]. Anxiety levels were cathegorised according to the scores obtained from the answers: low (20–34); moderate (35–49); high (50–64); very high (65–80). Physical activities during pregnancy were evaluated trough the Pregnancy Physical Activity Questionnaire (PPAQ) which evaluates 31 every-day physical activities (11 related to household/care-giving, 5 to occupational activities, 9 to sports/exercise, 3 to transportation, and 3 to inactivity) which were performed during the last month before consultation and the time invested in the reported activities. For each participant the number of minutes in each of the reported activities was multiplied by its metabolic equivalent (MET) intensity and summed to obtain a measure of the average weekly energy expenditure (MET-hrs/wk). The PPAQ questionnaire used was adapted from the study by Chasan-Taber et al. [20], for use in the Portuguese language in Brazil [21]. To minimise biases, the STAI and the PPAQ were completed by the participants themselves, since the study was not blinded to the evaluators. Pelvic floor muscle knowledge was evaluated using categorical questions elaborated for this purpose to asses if they had knowledge or had heard about pelvic floor muscles and its function. Any information given on these issues was considered a positive answer. Urinary incontinence was evaluated through a set of questions elaborated for this study to collect information on women’s urine leakage, either as a result of stress incontinence or incontinence associated with urgency. Because it was a pragmatic study, pelvic floor function was not evaluated. Participants who reported any involuntary loss of urine were considered incontinent [22]. Lumbopelvic pain self-reported by women and confirmed by the women indicating the localization of the pain on their own body and on a graphical representation of the human body and the perception of the intensity of the pain was evaluated using a 10 cm visual analogue scale (VAS) [23] on which the participants recorded the average of pain experienced over the preceding days. Neonatal wellbeing, evaluated using the 1^st^ and 5^th^ minute Apgar scores, and perinatal results were obtained from the participants’ medical records. To evaluate the adherence of the intervention group with the exercises to be performed at home, a diary was given to each woman to record the activities performed. The women were instructed to bring the diary back to the clinic on follow-up visits.

### Randomisation

Prior to randomisation, all the participants completed the STAI and the PPAQ, and their sociodemographic and obstetric data were collected. To guarantee that the allocation to groups remained concealed until women were admitted to the study; randomisation was done by opening a sealed, opaque, consecutively numbered envelope containing the information on the group to which the participant was being allocated in accordance with a previously prepared, computer-generated random sequence of numbers. The randomisation was 1:1, and the process and preparation of the envelopes containing the information were carried out by a person who was not directly involved with the study. Participants received only reimbursement of expenses related with participation in the study.

### Intervention

The women assigned to the control group participated in educational activities routinely offered at the prenatal clinics where they received prenatal care. These activities consisted of information provided by the nursing staff on breastfeeding, the signs and symptoms of labor and a visit to the delivery ward. During labor, at the maternity ward, non-systematic information on the use of non-pharmacological pain relief techniques was provided by trained physiotherapy, nursing and medical staff.

Women of the intervention group participated in the physical and educational activities of the BPP conducted in addition to routine activities offered at the prenatal clinic, on the same days of the prenatal visits, in order to minimize the difficulties women from low resource settings have to attend health education activities organized otherwise. During the meetings of approximately 50 minutes women performed non aerobic exercises of a protocol adapted for pregnancy and designed to attempt to reduce back pain, possibly to help venous return and to prevent UI and minimize anxiety (Table 1). At the first BPP meeting women received oral guidance on the awareness of pelvic floor muscles–they were instructed to contract the pelvic floor as if they were trying to avoid urination and focus on the sensation of the pelvic floor muscles during this movement and during relaxation after the contraction. Women who did not manage to perform this exercise were advised to try the contraction of the pelvic floor during urination, attempting to stop urine flow and informed that this exercise during urination was only for the identification of the sensation.

**Table 1:** Short description of the BPP exercises

**Stretching**	Head and neck; anterior, posterior and lateral trunk; lower limbs; active mobilization of the spine and pelvis in the position of all fours; lumbar traction
**Venous return**	Exercises with the lower limbs in the standing and lateral decubitus position
**Abdominal exercises**	Transverse abdominal muscle activation in both standing and all four positions
**PFMT**	Maximal rapid and sustained contraction on standing and sitting position
**Relaxation**	Training of breathing techniques for contraction control during labor; progressive relaxation techniques; massage; mentalization

During BPP meetings information was provided on the prevention of pain in pregnancy, the role of the pelvic floor in pregnancy, delivery and in the postpartum, the physiology of labor, breathing exercises for delivery, and non-pharmacological pain control techniques during labor. Participants received a guide with the exercises to be performed daily at home, consisting of: pelvic floor muscle training (PFMT) including rapid (30 times) and sustained maximal contractions (20 times holding for 10 seconds), stretching exercises to reduce back pain and exercises to improve venous return in the lower limbs. The women were also encouraged to practice aerobic exercise daily for at least 30 minutes and received written information based on the ACOG guidelines [2] regarding the warning signs that indicated that the exercise should be stopped.

The activities performed in the BPP were planned and structured specifically for the study and supervised by trained physiotherapists who worked exclusively for the study. The birth preparation programme meetings were held on the same days of prenatal visits on a monthly basis up to 30 weeks of pregnancy, fortnightly between 31 and 36 weeks of pregnancy and weekly from 37 weeks of pregnancy onwards. Meetings were either conducted as open group or individual sessions; the modality depended on the number of women present.

### Statistical analysis

Analysis was performed according to the group to which participants were assigned, on an intention-to-treat basis. To test the differences between the groups, the Mann–Whitney test and Student’s *t*-test were used for the continuous variables and the *χ*^2^ test and Fisher’s exact test for the categorical variables. For categorical dependent variables, risk ratios (RR) were calculated, together with their respective 95% confidence intervals (95%CI). For numerical dependent variables, mean differences and their respective 95%CI were also estimated. Significance was established at p<0.05.

## Results

**Figure 1:**
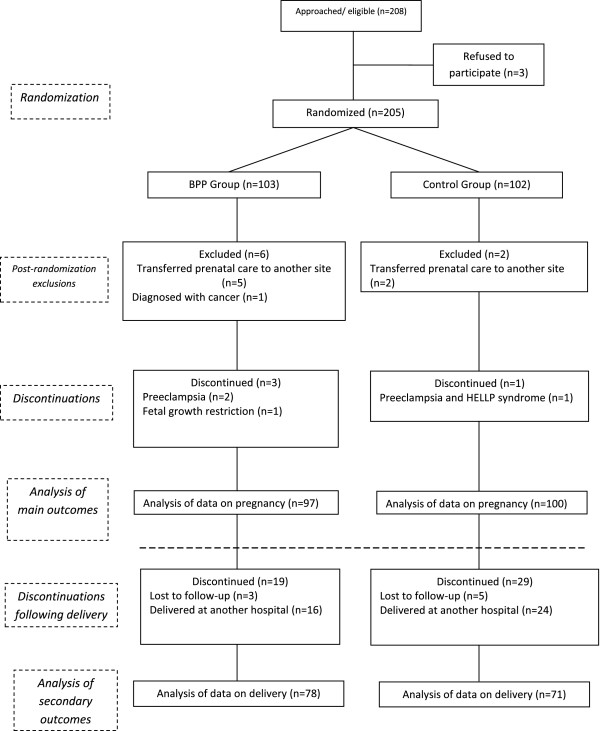
Flowchart of the allocation and follow-up of the participants in the study.

For the study, 208 eligible pregnant women were identified. Three women refused the invitation to participate and 205 women were randomised into the intervention and control groups. Eight women were excluded post-randomisation. A total of 197 women participated in the study, 97 allocated to the intervention group and 100 to the control group. Figure 1 shows randomisation process regarding the groups and the follow-up of the participants in accordance with the flowchart suggested by the Consolidated Standards of Reporting Trials (CONSORT) [24]. Apart from the 8 initial cases of exclusion following randomisation, the only data that were lost was secondary data referring to delivery and the newborn infant in 48 cases (24%) in which delivery occurred in other institutions. There was no difference between the groups regarding the sociodemographic and obstetric characteristics (Table 2). The women in the BPP intervention group participated in a median of 5 meetings (range 2 to 10 meetings).

**Table 2:** Socio demographic and obstetric characteristics at baseline evaluation

**Characteristics**	**BPP group (n=97)**	**Control group (n=100)**
Age (mean±sd)	22.9 ± 4.6	22.9 ± 5.1
Gestational age (mean±sd)	20.7 ± 1.8	20.4 ± 2.0
Body mass index (mean±sd)	25.4 ± 5.0	25.2 ± 5.3
	**N (%)**	**N (%)**
*Education*		
Primary	16 (16.5)	19 (19.0)
Secondary	64 (66.0)	64 (64.0)
University	13 (13.4)	17 (17.0)
Technical	4 (4.1)	0 (0.0)
Steady partner	77 (79.4)	78 (78.0)
Planned pregnancy	51 (53.1)	47 (47.0)
Practiced physical activity prior to pregnancy	25 (25.8)	31 (31.0)
Knowledge of pelvic floor muscles	23 (23.7)	13 (13.0)
Urinary incontinence prior to pregnancy	17 (17.5)	18 18.0

All values are not statistically significant.

When compared baseline data, a significant reduction was reported in the number of complaints of UI by the women in the BPP group (41%), whereas in the control group the number of complaints increased at the final evaluation (68%) (Table 3). The number needed to treat (NNT) was 5.1 at the intermediate evaluation and 3.7 at the final evaluation. There was no difference between the groups regarding to the prevalence or to the intensity of lumbopelvic pain throughout pregnancy (Table 4). In addition, the use of medication to control pain was similar in both groups at the three evaluation times (data not shown).

**Table 3:** Reported urinary incontinence by group and the risk of developing urinary incontinence

	**BPP Group N (%)**	**Control Group N (%)**	**RR (95%CI)**	**n**
Baseline	52 (53.6)	53 (53.0)	1.01 (0.78–1.31)	197
Intermediate evaluation	38 (42.7)	46 (62.2)	**0.69 (0.51–0.93)**	164
Final evaluation	35 (41.2)	52 (68.4)	**0.60 (0.45–0.81)**	161

Values in bold mean they are statistically significant.

**Table 4:** Occurrence of lumbopelvic pain and the respective pain scores of the intervention and control group

**Prevalence of pain**	**BPP group N (%)**	**Control group N (%)**	**RR (95%CI)**	**n**
*Lumbar pain*				
Baseline	57 (58.8)	65 (65.0)	0.90 (0.73–1.13)	197
Intermediate evaluation	53 (59.6)	47 (63.5)	0.94 (0.74–1.20)	164
Final evaluation	54 (63.5)	48 (63.2)	1.01 (0.79–1.27)	161
*Pelvic pain)*				
Baseline	14 (14.4)	15 (15.0)	0.96 (0.49–1.89)	197
Intermediate evaluation	24 (27.0)	15 (20.3)	1.33 (0.75–1.34)	164
Final evaluation	24 (28.2)	21 (27.6)	1.02 (0.62–1.68)	161
**Intensity of pain**	**VAS (cm)**	**VAS (cm)**	**Mean difference (95% CI)**	
*Lumbar pain* (mean±sd)				
Baseline	4.7 ± 2.7	4.5 ± 2.2	0.23 (-0.64–1.09)	122
Intermediate evaluation	5.1 ± 2.3	5.1 ± 2.5	0.08 (-0.86–1.03)	99
Final evaluation	5.1 ± 2.3	4.8 ± 2.5	0.34 (-0.61–1.28)	102
*Pelvic pain* (mean±sd)				
Baseline	3.8 ± 2.1	4.7 ± 2.4	-0.9 (-2.49–0.78)	29
Intermediate evaluation	4.9 ± 2.7	5.4 ± 2.3	-0.47 (-2.12–1.19)	39
Final evaluation	5.5 ± 2.9	5.9 ± 2.8	-0.38 (-2.09–1.33)	44

**Figure 2:**
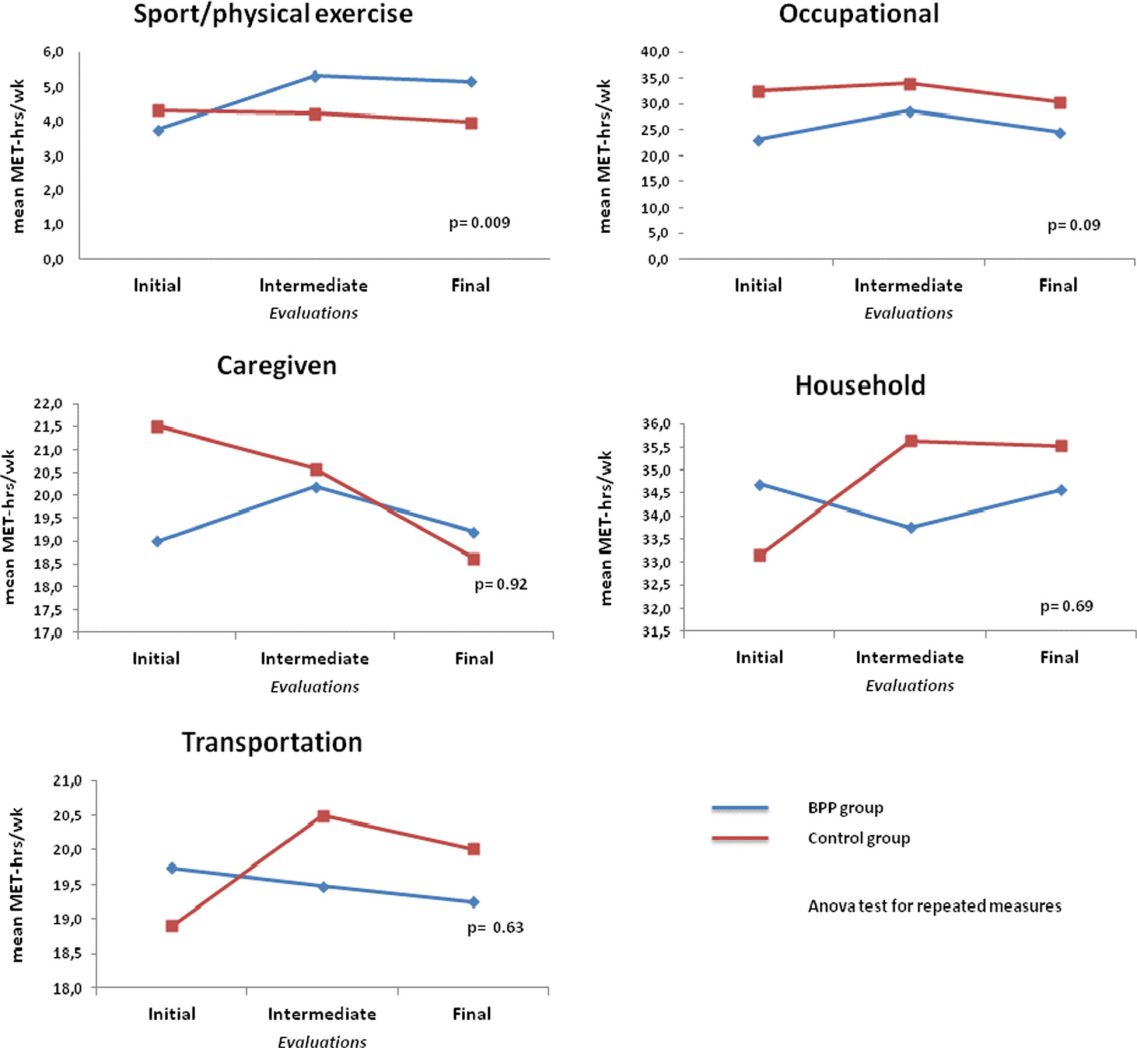
Comparison of energy expenditure per week among groups.

Anxiety level was similar in both groups throughout pregnancy, being low/moderate (scores between 20 and 49) in the majority of women at the three evaluations. The Table 5 shows the percentages of the women who presented high and very high anxiety levels. Data related to the physical activities evaluated using the PPAQ, showed a difference between the groups regarding the energy expenditure of physical exercise, with increased (initial to final evaluation) in the BPP (1.4 MET-hrs/wk) and decreased in the CG (-0.3 MET-hrs/wk), with a significant difference between groups (p=0.009; Anova test). There was no difference between the groups regarding the other types of physical activity (Figure 2). The median increase in maternal weight between baseline and the final evaluation was similar in both groups, 8,5 kg (interquartile range–Q1: 6,2 kg; Q3: 11,8 kg) in the BPP group and 7,9 kg (Q1: 6,4 kg; Q3: 10,6 kg) in the control group (p=0.81, Mann–Whitney test; data not shown). Analysis of the participants’ adherence with the exercises to be performed at home was unfeasible because the women either failed to complete the exercise diary or failed to bring it with them on their return to the clinic. No adverse events associated with the exercise were reported by the participants. Regarding the analysis of the perinatal data, no difference was found between the groups, except for length of delivery (Table 6).

**Table 5:** High and very high anxiety level of the intervention and control group

	**BPP group N (%)**	**Control group N (%)**	**RR (95%CI)**	**n**
*STAI TRAIT)*				
Baseline	33 (34.0)	26 (26.0)	1.30 (0.84–1.99)	197
Intermediate evaluation	17 (19.1)	20 (26.7)	0.72 (0.41–1.27)	164
Final evaluation	18 (21.2)	20 (26.3)	0.80 (0.46–1.40)	161
*STAI STATE)*				
Baseline	18 (18.8)	21 (21.0)	0.89 (0.51–1.57)	197
Intermediate evaluation	16 (18.0)	16 (21.3)	0.84 (0.45–1.57)	164
Final evaluation	16 (18.8)	14 (18.4)	1.02 (0.53–1.95)	161

**Table 6:** Obstetric, perinatal and neonatal data of the intervention and control group

	**BPP Group (mean±sd)**	**Control Group (mean±sd)**	**Mean difference (95% CI)**
Cervical dilation at admission to hospital (cm)	3.5 (±2)	3.0 (±1.9)	0.55 (-0.13–1.23)
Duration of active phase (min)	284.5 (±175)	254.2 (±139.4)	30.3 (-40.9–101.4)
Duration of delivery (min)	29.2 (±23.3)	19.7 (±13)	**9.48 (0.32–18.64)**
Cervical dilation at analgesia (cm)	8 (±1.2)	6.6 (±3.2)	1.47 (-1.74–4.68)
	**N (%)**	**N (%)**	**RR (95%CI)**
Gestational age at delivery ≥37 weeks	67 (90.5)	64 (92.8)	0.98 (0.88–1.08)
Vaginal delivery	44 (57.9)	38 (53.5)	1.08 (0.81–1.44)
1^st^ minute Apgar ≥7	70 (93.3)	63 (92.7)	1.01 (0.92–1.10)
5^th^ minute Apgar ≥7	75 (100)	67 (98.5)	1.01 (0.99–1.04)
Birthweight ≥2500g	70 (92.1)	64 (94.1)	0.98 (0.90–1.07)

Values in bold mean they are statistically significant.

## Discussion

This study evaluated a birth preparation programme linked to women’s visits to the clinic for prenatal care that included various interventions: general supervised exercise, information on performing aerobic and local exercises at home and educational activities. The main findings showed that a systematically performed programme with clear objectives exerts a positive effect by reducing complaints of UI and increasing the practice of physical exercise throughout pregnancy.

In relation to the prevention and control of UI during pregnancy, these findings are in agreement with the results of a systematic review [10] that showed that nulliparous women are able to avoid UI in pregnancy by performing PFMT. Maybe, the fact that women in the BPP group had received information regarding pelvic floor muscle and PFMT resulted in increased awareness and consequently in reduced urinary leakage [25]. Furthermore, PFMT was given at the BPP meetings and the importance of continuing to practice these exercises at home was emphasized.

Regarding the practice of exercise, prevalence studies showed that women do not tend to comply with the guidelines recommended by ACOG for the practice of exercise [2], perform little exercise during pregnancy and the practice of exercise tends to diminish as pregnancy progresses [26-30]. In accordance with the results of the present study, the energy expenditure improvement with the practice of physical exercise during pregnancy may be associated with encouragement and guidance. Although they had maintained a moderate level of exercise intensity (3.0–6.0 METs) [20], the women in the control group, who received neither guidance nor encouragement, had a decrease in energy expenditure with the practice of physical exercise during pregnancy.

On the other hand, the BPP had no effect on relieving or preventing lumbopelvic pain. Other studies have shown that specific exercises to reduce pain in the lumbopelvic region were effective when the exercise was supervised and practiced once a week or once a fortnight [31-34]. In the present study, supervised practice occurred only on the days of prenatal medical visits, which were scheduled monthly throughout most of the pregnancy and fortnightly between 31 and 36 weeks of pregnancy and weekly from 37 weeks of pregnancy onwards. In addition, the exercises that the women were instructed to perform at home consisted of only two types of stretching exercise for the lumbopelvic region, which, in our opinion, are those safest and easiest to carry out unsupervised. On the other hand, in previous studies [32,33], the number of exercises that the women were counseled to perform at home for this purpose was greater. These results lead us to believe that this type of discomfort during pregnancy requires greater attention, with a need for more frequent supervised interventions, a greater number of specific exercises or the practice of different types of exercise at home. It was not possible to evaluate adherence to home exercises for pain in the present study.

At the end of pregnancy, anxiety levels may increase as labor approaches [35]. Nevertheless, in this study anxiety remained low or moderate in both groups. This may be due to the fact that the women participated in routine counseling groups throughout their prenatal care at which timely information was provided on the signs and symptoms of labor and visits were made to the delivery ward. Furthermore, the study population consisted of low-risk pregnant women with access to prenatal care, which may have contributed to their low levels of anxiety.

One limitation of this study was that the attempt to evaluate adherence by asking the women in the study to complete an exercise diary was not successful since women failed to complete their diaries and consequently it was not possible to perform an analysis of adherence. Also another possible limitation was the loss to follow-up due, in some cases, to the fact that some participants delivered their babies at other facilities and in other cases to the loss of contact with some of the participants, which hampered evaluation of the secondary data. Another limitation may lay on the fact that the study was not blinded, what could have resulted in women giving “right answers” to questions regarding urinary incontinence and practice of physical exercise as a courtesy bias. However, if this was the case, the same could be expected for answers concerning lumbopelvic pain and anxiety, but this did not happen at all.

The results of this study may contribute towards improving birth preparation programmes; however, further studies are required to establish the most effective techniques for reducing lumbopelvic pain. Further evaluation also needs to be made of the ideal number of meetings and how frequently they should take place for a programme including different types of interventions.

## Conclusions

This BPP was effective in preventing and controlling UI and in encouraging women to exercise during pregnancy, while preserving maternal and fetal health; however, the program failed to control lumbopelvic pain, and had no effect on perinatal outcome.

### Details of ethics approval

This study was approved by the Institutional Review Board of the School of Medicine, State University of Campinas (UNICAMP) under registration number 407/2008 on 24 June 2008. The study was also registered at clinicaltrials.gov (NCT01155804).

## Abbreviations

RCT: Randomized controlled trial; BPP: Birth preparation program; CG: Control group; RR: Risk ratio; CI: Confidence interval; ACOG: the American College of Obstetricians and Gynecologists; CAISM: Women’s integral health care hospital; UNICAMP: University of Campinas; PPAQ: Pregnancy physical activity questionnaire; STAI: State-trait anxiety inventory; PFMT: Pelvic floor muscle training; MET: Metabolic equivalent.

## Competing interest

The authors declare that they have no competing interests.

## Authors’ contributions

MAM participated in designing the research project, was involved in the data collection, in the interpretation of the results and writing of the manuscript. MYM participated in designing the research project, interpreting the results and preparing the manuscript. JGC contributed to the final editing of the research project, interpretation of the results and preparation of the manuscript. All authors read and approved the final manuscript.

## Pre-publication history

The pre-publication history for this paper can be accessed here:

http://www.biomedcentral.com/1471-2393/13/154/prepub
